# Identification of cancer-related genes *FGFR2* and *CEBPB* in choledochal cyst via RNA sequencing of patient-derived liver organoids

**DOI:** 10.1371/journal.pone.0283737

**Published:** 2023-03-30

**Authors:** Yongqin Ye, Vincent Chi Hang Lui, Rosana Ottakandathil Babu, Zhongluan Wu, Weifang Wu, Patrick Ho Yu Chung, Kenneth Kak Yuen Wong, Bin Wang, Paul Kwong Hang Tam

**Affiliations:** 1 Department of Surgery, School of Clinical Medicine, The University of Hong Kong, Hong Kong, China; 2 Faculty of Medicine, Macau University of Science and Technology, Macau SAR, China; 3 Department of General Surgery, Shenzhen Children’s Hospital, Shenzhen, Guangdong, China; 4 Dr. Li Dak-Sum Research Centre, The University of Hong Kong, Hong Kong, China; 5 Department of Surgery, University of Hong Kong-Shenzhen Hospital, Shenzhen, Guangdong, China; Texas A&M University, UNITED STATES

## Abstract

**Background:**

Choledochal cysts (CC) are congenital bile duct anomalies with 6–30% risk for developing bile duct cancer. However, the molecular mechanisms underlying cancer risk of CC are unknown. We sought to identify the gene expression changes underlying the cancer risk of CC patients.

**Methods:**

Liver organoids (n = 51) were generated from liver/bile duct biopsies of CC (n = 7; type I) and hepatoblastoma (n = 5; HB: non-tumor & tumor) for RNA sequencing. Bioinformatics analysis was conducted to identify differentially expressed cancer-related genes in CC and controls. We compared CC with non-cancerous and cancerous controls, normal adjacent non-tumor region of hepatoblastoma (HB) liver as non-cancerous control and tumor region as non-CC cancer control (HB-tumor). Reverse transcription real-time quantitative PCR (RT-qPCR) verification and immunohistochemistry of selected genes was conducted in additional CC and HB liver biopsies.

**Findings:**

HB non-tumor and HB tumor organoids displayed distinct gene expression profiles. Expression profiling separated CC organoids into two clusters, one overlapping with HB non-tumor and the other one with HB tumor organoids. Genes selected based on their log2FoldChange values for RT-qPCR verification in 31 CC and 11 HB non-tumor liver tissues revealed significantly elevated expression of *FGFR2* in 7 and *CEBPB* in 2 CC liver tissues (CC vs HB: 4.082 vs. 0.7671, *p*<0.01; 2.506 vs. 1.210, *p*<0.01). Distinctive positive staining in bile ducts were seen in CC, HB tumor and non-tumor liver tissues for FGFR2 and CEBPB. Percentages of CEBPB-immuno-positive or FGFR2-immuno-positive bile duct cells in CC and HB-tumor liver were higher than that in HB non-tumor liver.

**Interpretation:**

The study identified dysregulated genes related to cancer pathways in CC patients suggesting cancer risk. The findings suggest that the elevated expression of *FGFR2* and *CEBPB* in liver may contribute to cancer development in CC patients.

## Introduction

Choledochal cysts (CCs) are characterized by congenital dilation of the extra-hepatic and/or intra-hepatic biliary tree, with an incidence of 1 in 1,000 live births in Asian population [1]. With an increasing popularity of fetal ultrasound after 2000s, more than 90% of choledochal cyst can be detected antenatally [2]. Post-natally, affected patients can remain asymptomatic for many years but can also suffer from jaundice, cholangitis, pancreatitis and intra-abdominal abscess. In view of these potential complications, cyst excision is necessary even for asymptomatic cases. Another reason to support the radical surgery is the associated cancer risk [3]. It has been known for long that untreated CCs are prone to the development of cancer with a risk ranging from 2.5 to 26% [4–7]. Even worse, the risk of cancer persists despite complete cyst removal [8]. The cumulative incidences of bile duct cancer at 25 years after complete cysts excision can be as high as 11.3% [9]. While this association between CC and bile duct cancer is supported by large-scale clinical studies, only a scarcity of reports on the etio-pathogenesis and molecular pathway that account for the malignant transformation are available [8].

Two main theories have been proposed for CC, the “obstructing segment” hypothesis and the “pancreatic reflux” hypothesis [10, 11]. Both theories suggest the presence of structural anomalies during the hepatobiliary-pancreatic development during embryonic stages. A recent “trio-based” exome-sequencing study which has identified damaging de novo variants in several evolutionarily constrained genes associated with human developmental disorders further supports the presence of hepatobiliary-pancreatic developmental anomalies in CC [12]. The same report also revealed that CC patients have an excess of de novo variants in cancer-related gene. However, it is unknown if CC livers display cancer-related transcriptome signature, which may explain a higher risk of bile duct cancer.

Bile duct cancer is a malignancy that arises from the intra-hepatic or extra-hepatic bile ducts. Although bile duct cancer displays histological and molecular features of an adenocarcinoma of biliary epithelial cells, the cellular origin is unknown. It has been suggested that bile duct cancer may arise from hepatic stem cells (HSC) that give rise to both hepatocytes and cholangiocytes [13–15]. Chronic inflammation and impaired bile flow cause damages to cholangiocytes and are thought to play a role in the HSC development from early hyperplasia and metaplasia, through dysplasia to carcinoma [16–18].

While bile duct cancer is extremely rare in children, Hepatoblastoma (HB) is the most common childhood liver cancer that accounts for over 90% of malignant liver tumors in children younger than 5 years of age [19]. Hepatocytes derived from the HSC of HB tumor recapitulated the tumor architecture, mutational profile, gene expression patterns of patient’s tumors [20]. HB tumor has also been shown to contain tumor-like cholangiocyte progenitors [21], suggesting but not proven, that HB tumor region HSC-derived cholangiocytes also exhibit cancer features.

Single cell RNA sequencing analysis and immunofluorescence analysis have indicated that liver tissue-derived organoids are consisted of HSC, HSC-derived hepatocytes, and HSC-derived cholangiocytes as a major cell type, and are good human proxy for modelling bile duct diseases [22–25], we employed liver tissue-derived organoids in this study to investigate if HSC-derived cholangiocytes in CC exhibit cancer features by comparing with HB non-tumor and HB tumor. We grew liver organoids from CC liver/bile duct biopsies and HB liver biopsies (tumor and adjacent non-tumor parts), followed by RNA sequencing. The HB adjacent non-tumor is represented as HB, while the HB region with tumor is represented as HB-tumor/HBT in the analysis. We found that CEBPB and FGFR2 were differentially elevated in the organoids and bile duct cells of CC patients and HB tumor region, and that elevated expression of FGFR2 and CEBPB may contribute to malignant transformation of CC.

## Methods

### Liver and bile duct tissues

The liver tissues were obtained from tumor and non-tumor margin of hepatoblastoma (HB); liver and/or bile duct biopsies of choledochal cysts (CC) patients. These CC patients were all classified as type 1. All tissues were obtained during operations with full written informed consent from parents or patients, and the study was approved by Hong Kong West Cluster-Hong Kong University Cluster Research Ethics Committee/Institutional Review Board (UW 16–052). Clinical information of the HB and CC patients recruited in the current study were depicted as shown in S4 and S5 Tables.

### Generation of human liver organoids

Liver organoids were generated from liver/bile duct biopsies following our previously published protocol [24]. Briefly, liver/bile duct biopsy was minced and digested in 5 ml of digestion medium in gentleMACS-C Tube (Multi Tissue Dissociation Kit 1), filtered (70 μm and 30 μm) and sorted for cholangiocyte progenitors by using human CD326 (EpCAM) magnetic beads. CD326 positive cells were mixed with Matrigel (50,000 cells in 50 μl) and added to each well of a four-well culture plate (Nunc™ 4-Well Dishes). After Matrigel solidification, organoid medium was added, and medium was changed every three days.

### RNA sequencing (RNA-seq) analysis of organoids

Organoids were retrieved from Matrigel to individual (one organoid per tube). Organoids lysis, RNA extraction, reverse transcription, amplification and library construction were performed using single cell RNA-seq technology (Smart-seq 2.0) [26] with minor modifications [24]. Libraries were sequenced by pair ends of 100 base pairs on an Illumina HiSeq 2500 system. All transcriptomic data have been uploaded to NCBI Sequence Read Archive (SRA), the transcriptomic data can be found with accession number: PRJNA549557.

### Bioinformatic analysis

The bulk RNA-seq data of organoids from HB patients (tumor and non-tumor organoids) and CC patients were subjected to bioinformatics analysis to identify transcriptome signatures of HB tumor organoids and CC organoids. The bulk RNA-seq reads were subjected to quality check and bioinformatics analysis following our previously published analysis pipeline for the identification of differentially expressed genes between different groups [24]. In brief, all the samples showed an overall >80% alignment with human reference. Aligned reads per gene counting and differential expression analysis were performed using HTSeq version 0.11.17. Normalization and identification of differentially expressed genes (DEGs) were done using R Limma-voom algorithm8 and DESeq2 version 1.18.9. Cut-off criteria to filter out low expression genes were CPM threshold value of 1 or log-CPM value of 0 using limma-voom. DEGs were taken with cut-off of adjusted p-value cut-off of 0.05 and log2FoldChange of 0.5 for all analysis. PCA plots were produced using pcaExplorer version 4.2 [27]. TSNE plots were generated directly for normalized counts with default parameters using Rtsne version 0.15 [28]. Heatmap with k-means clustering algorithm [29, 30] was applied to identify distinct expression pattern of genes. The Gene Ontology and pathway enrichment analysis were performed using the ClusterProfiler R package [31]. The annotation Dbi R package org.Hs.eg.db was used to map gene identifiers. Pathway analysis was also performed for each cluster of dysregulated genes by using the Gene Ontology database [32], the David Web Tools [33] and PANTHER (http://www.pantherdb.org/) using default parameters. The results were visualized using the R packages clusterProfiler [31] and ggplot2 [34].

### RT-qPCR (reverse transcription-qualitative PCR) analysis

While the dysregulated genes in CC from comparisons showed significantly enriched cancer related pathways using KEGG pathway analysis, top ten DEGs that were found to be highly regulated in some CC organoids and HBT organoids compared to HB non-tumor organoids based on log2FoldChange values were selected for further verification in CC liver tissues. Total RNA was extracted from liver tissues of type 1 CC and HB using RNeasy® Mini RNA Extraction Kit (Qiagen), and concentration of RNA was measured using Nanodrop. Then the extracted RNA underwent genomic DNA elimination and reverse transcription with PrimeScript RT reagent kit with gDNA Eraser (PrimeScript™ RT reagent Kit with gDNA Eraser, Perfect Real Time, Cat. # RR047A; Takara) following the manufacturers’ protocols. Real-time PCR analysis was performed on 7900 Fast Real-Time PCR system using iTaq Universal SYBR Green Supermix (Bio-Rad) using specific primers (S1 Table). Relative expression levels of these genes were determined using GAPDH as an internal reference and the 2^–ΔΔCt^ method. The qPCR reaction protocols were as the followings: 10 μl of iTaq Universal SYBR Green Supermix (2x), 0.5 μl of forward primer, 0.5 μl of reverse primer, 1 μl of cDNA template (10 ng/ul), and 8 μl of H_2_O. The reaction program was performed at 95˚C DNA denaturation for 2 minutes, followed by 40 cycles at 95˚C denaturation for 1 second and at 60˚C annealing/extension for 30 seconds. An additional melt curve stage was carried out at 95°C for 15 seconds and 54°C for 1 minute, followed by dissociation step at 95°C for 15 seconds.

### Immunohistochemistry staining

To better understand the expression pattern of these genes in liver tissue, we performed immunohistochemistry (IHC) on a separate cohort of CC (n = 35) and HB (non-tumor; n = 10) patients’ livers. Liver tissues were fixed in 4% paraformaldehyde/phosphate buffer saline pH 7.0 (PFA/PBS) for 48 hours at 4°C, dehydrated in a graded series of alcohol, cleared in xylene before being embedded in paraffin. Liver sections (6 μm) were prepared and mounted on microscope glass for immunostaining. Sections were dewaxed in xylene, then rehydrated in a graded series of alcohol, and finally in distilled water. Antigen retrieval was performed in 10 mM Na Citrate pH6.0 at 95°C for 10 minutes. Endogenous peroxidase activity was blocked with Hydrogen Peroxide Block (3% H_2_O_2_ in methanol) at room temperature for 10 minutes. After washing in PBS, sections were blocked with PBS-T (PBS with 0.1% Triton) supplemented with 1% Bovine Serum Albumin (BSA) at room temperature for 30 minutes. Primary incubation was performed with primary antibody diluted in PBS-T/BSA at 4°C overnight. After washing (3x5 minutes each) in PBS, sections were incubated with secondary antibody (S2 Table) at 37°C for 1 hour. After PBS washing (3x5 minutes each), signal was developed with DAB kit. Sections were counterstained with haematoxylin for 30 seconds, washed in running tap water for 2 minutes, incubated in Scott’s tap buffer for 30 seconds before finally washed in tap water for 10 minutes. Section were dehydrated in a graded series of alcohol, cleared in xylene and then mounted in Permount medium. Images were taken with a Nikon Eclipse E600 microscope mounted with a Nikon Digital Camera DXM1200F.

### Statistical analysis

All results were presented as Mean±Standard error of the mean (SEM) if normally distributed, otherwise Median. Statistical comparisons were evaluated using Mann Whitney U test or unpaired Student’s t-test. The correlation between clinical parameters and immuno-reactivity was performed using Pearson. All statistical analyses were performed with GraphPad Prism 6.0. A *p*-value <0.05 was considered statistically significant.

## Results

### Establishment of liver organoids from choledochal cyst and Hepatoblastoma tumor and non-tumor liver tissues

**Fig 1:**
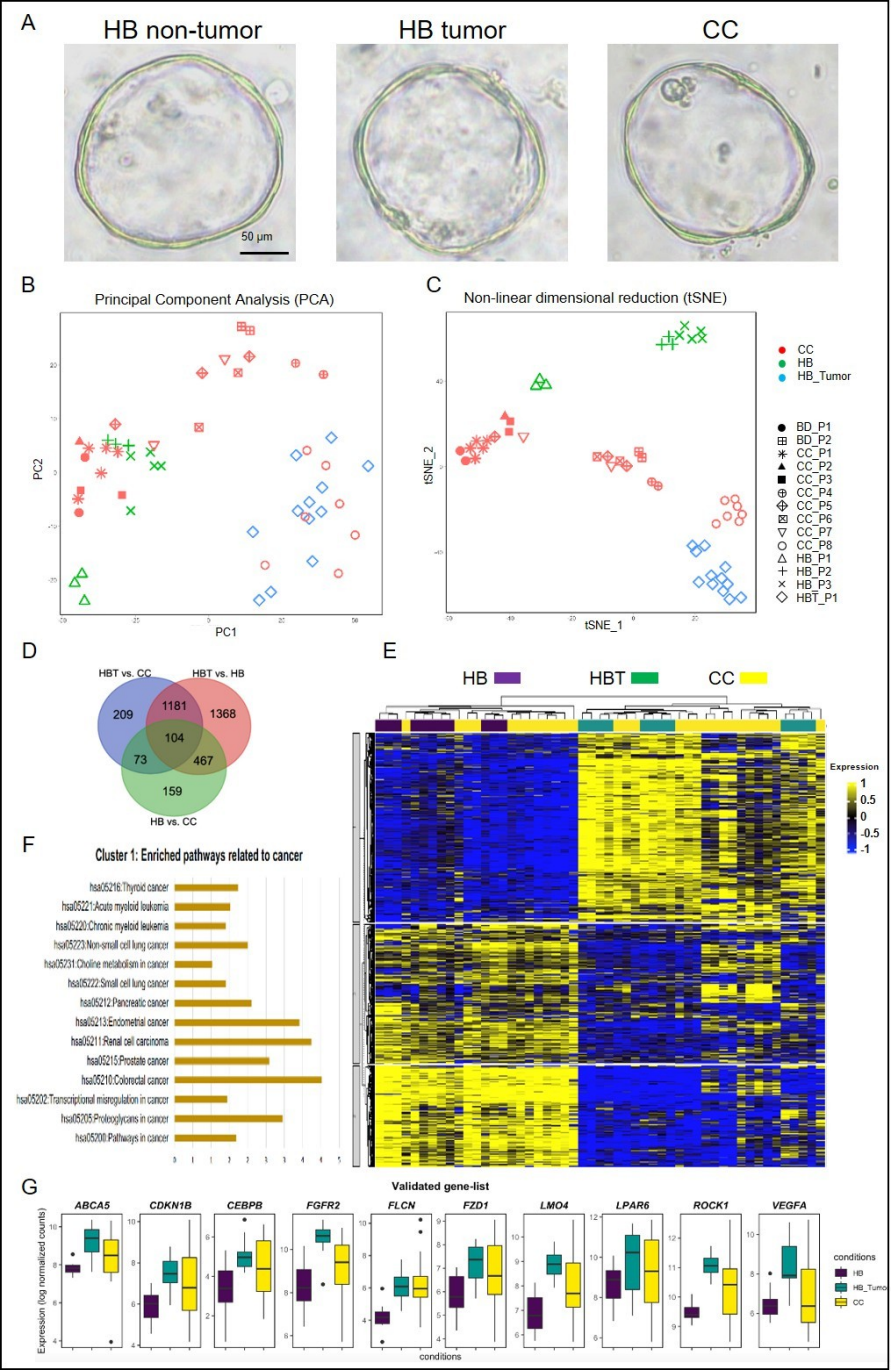
Expression levels for dysregulated cancer-related genes from patient derived organoids. (A) Pictures of P0 cholangiocyte organoids from hepatoblastoma (HB) non-tumor and tumor livers; choledochal cyst (CC) livers/bile ducts. (B) Principal Component Analysis (PCA) analysis showing first two PCs capturing highest variance, 69% (dimension 1: 60% + dimension 2: 9%) in expression data; each dot represents an organoid. (C) Non-linear dimensional reduction (TSNE) showing clustering of patient derived organoid. Each dot represents an organoid; CC, HB and HBT organoids were indicated with different colours; and different shapes indicated organoids from patient with I.D.. CC organoids clustering close with HBT organoids were enclosed with broken blue line. (D) Heatmap clustering shows total differentially expressed genes and grouping of CC samples with HB and HB-Tumour. (E) Significantly enriched cancer related pathways for DE genes from cluster 1, using KEGG pathway analysis. (F) Expression levels of the top 10 DE cancer-related genes in HB and CC organoids.

Following the published protocol [23, 24], we established 51 liver organoids from patient liver/bile duct biopsies at the time of the surgical procedure from hepatoblastoma (HB) and choledochal cyst (CC) for the study. In total 23 organoids were established for HB (12 organoids from 1 patient with HB-tumor and 11 organoids from 3 patients for HB non-tumor regions). In total, 28 organoids for CC were derived from liver tissue (24 organoids from 8 patients) as well as bile duct (4 organoids from 2 patients). HB-tumor, non-tumor and CC organoids appeared in culture in 3–5 days, grew into large, well-expanded cystic structures with a single layer of epithelial cells in 15–20 days (Fig 1A). The P0 (Passage 0) CC and HB organoids were retrieved from the day-20 culture and processed for RNAseq to investigate the transcriptomes of CC, HB tumor and non-tumor organoids. Single cell sequencing of HB-normal organoids produced 5885 cells in total. The cell populations in single-cell derived clusters of HB-normal organdies were analysed and visualized by uniform manifold approximation and projection (UMAP), partitioning the cells into three clusters (S1 Fig). The common cholangiocyte markers epithelial cell adhesion molecule (EPCAM) and cytokeratin 19 (KRT19) were highly expressed in all the clusters, indicating the heterogeneity of cholangiocytes in these organoids was relatively low. The top markers for each clusters used to identify these cell-types are provided in S1 Fig.

### RNAseq revealed distinct transcriptomes between HB tumor and non-tumor organoids

We subjected 51 organoids from HB patients (tumor and non-tumor organoids) and CC patients to bulk transcriptome analysis. Principal Component Analysis (PCA) analysis revealed that the transcriptomic signature of HB tumor (HBT) organoids was distinct from the surrounding non-tumor liver tissue derived organoids (HB) (Fig 1B). In line with PCA analysis, non-linear dimensional reduction (tSNE) clustering also revealed significant transcriptomic differences in HB-tumor (HBT) and HB-non-tumor (HB) region as they formed two distinct clusters (Fig 1C, S2C Fig). The differential expression of cancer related genes in HB-tumor when compared with HB non-tumor (HB) suggests that the signature of HB-tumor and non-tumor liver tissue (HB) were different (S2C and S2F Fig). Differential expression results, showed transcriptome of HB-tumor organoids was distinct from HB non-tumor organoids with 3120 DE genes (S2F and S2G Fig). The expression profiles of dysregulated genes in HB-tumor are enriched in cancer related pathways such as prostate cancer, renal carcinoma, tight junction and chemical carcinogenesis pathways (S2J Fig), this suggest that the adjacent HB non-tumor liver is devoid of tumor. Comparison of HB non-tumor organoids with HB-tumor organoids (HB-tumor vs. HB) elucidate transcriptomic differences between these two regions of liver tissue. In line with the finding that HB-tumor liver contained tumor-like cholangiocyte progenitors [21], organoids derived from HSC of HB-tumor liver exhibited transcriptome with signature of cancer related pathways, which suggested that HB tumor region HSC also displayed cancer features.

### Clustering of CC organoids along with HB tumor and non-tumor organoids shows heterogeneity in CC patients

PCA analysis of 51 organoids (Fig 1B, S3A Fig) also revealed that organoids from two CC patients (CC_P8, CC_P4) clustered close to HB-tumor (enclosed with broken line) while others clustered towards HB-normal. TSNE clustering further showed CC liver- and bile duct-derived organoids from two more CC patients (CC_P8, CC_P4, BD_P2, 4 organoids from CC_P6 and one organoid from CC_P7) were clustering close to HB-tumor (enclosed with broken line) indicating transcriptomic similarity of these CC-organoids to tumor derived organoid transcriptomic signatures (Fig 1C, S3B Fig). The heterogeneity in CC patient derived organoids and tendency to cluster together with HB-tumor could be due to presence of tumor-like cholangiocyte progenitors, which has been reported in HB tumor [21].

### Organoids from CC showed tumor associated transcriptome signatures

To identify relative expression profiles of CC, we compared CC with both non-cancerous control (adjacent non-tumor region of HB) and non-CC cancer control (HB-Tumor). The differential expression results of HB non-tumor organoids with CC organoids (HB vs. CC) revealed several genes related to renal, prostate, endometrial carcinoma pathways being dysregulated in CC (S2A, S2D and S2H Fig). The comparison of HB-tumor organoids with CC organoids (HBT vs. CC) also suggested the dysregulation of genes related to pancreatic, chemical carcinogenesis pathways related to cancer being enriched in CC (S2B, S2E and S2I Fig). These results suggested that cancer related metabolic pathways are enriched in CC patients.

Differential expression comparison between HB non-tumor, HB-tumor and CC organoids revealed 3561 dysregulated genes. There were 1567 DE genes in HBT vs. CC and 803 DE genes in HB vs. CC comparison. As seen in PCA/tSNE, CC organoids from 5 patients were clustering together with HB-tumor in heatmap clustering (Fig 1D). The dysregulated genes in CC from comparisons (HBT vs. CC and HB vs. CC) showed enrichment of pathways related to cancer (Fig 1E, S2H, S2I and S3D Figs). The genes related to cancer including *ABCA5*, *CDKN1B*, *CEBPB*, *FGFR2*, *FLCN*, *FZD1*, *LMO4*, *LPAR6*, *ROCK1* and *VEGFA* were highly expressed in CC organoids and were also upregulated in HB-tumor organoids when compared to HB non-tumor organoids. Comparison of their expression levels are displayed in (Fig 1F), these genes could help understand the tumor related nature of CC patients as they are seen upregulated in both CC and HB-tumor.

### *FGFR2* and *CEBPB* expressions were significantly elevated in CC livers

**Fig 2:**
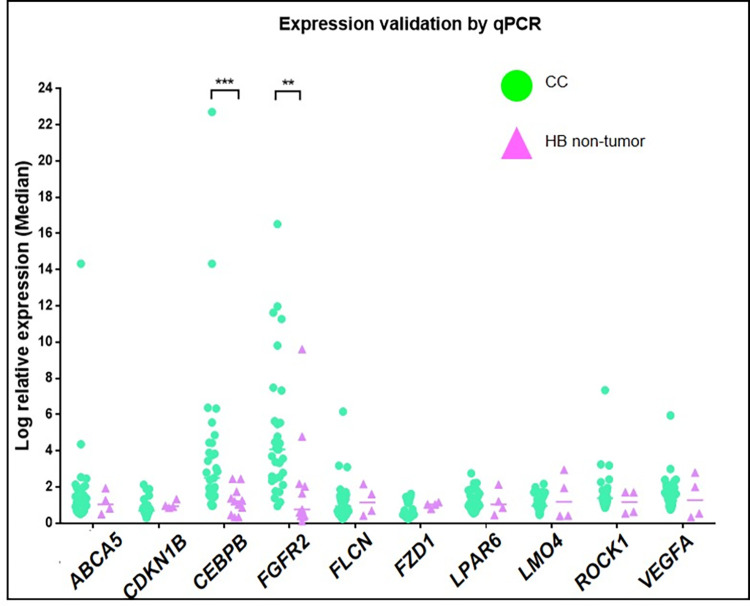
Elevated *FGFR2* and *CEBPB* expression in CC livers. Reverse transcription real-time quantitative polymerase reaction (RT-qPCR) validation of the DE genes in CC and HB livers. Total RNA was extracted from CC livers (n = 31) and HB non-tumor liver region (n = 11). Relative expression levels of the DE genes were determined using GAPDH as an internal reference and the 2^–ΔΔCt^ method. **, p<0.01.

Above mentioned DE cancer-related genes were highly expressed in CC and HB-tumor liver organoids compared to HB non-tumor organoids. To validate the observations, total RNAs of a new cohort of CC (n = 31) and HB (n = 11) non-tumor livers were prepared for reverse transcription quantitative polymerase chain reaction (RT-qPCR) analysis. The qPCR results showed that the mRNA expression of *FGFR2* and *CEBPB* were significantly upregulated in CC samples compared to that in HB, while some other genes from the analysis results had a varied expression level in CC samples vs. HB and the differences did not reach a statistical significance (Fig 2; Table 1).

**Table 1:** Mann Whitney U test on the expression of DE genes in CC and HB livers.

Markers	Expression Level (Median)		*U*-value	*P*-value	Significance
	CC (Experiment)	HB (non-tumor)			
*ABCA5*	1.208	1.047	47.00	0.4410	NS
*CDKN1B*	0.6940	0.9388	28.00	0.0824	NS
*CEBPB*	2.506	1.255	49.00	0.0002	S
*FGFR2*	4.082	0.7671	61.00	0.0011	S
*FLCN*	0.9394	1.149	58.00	0.8612	NS
*FZD1*	0.4834	1.045	34.00	0.1596	NS
*LPAR6*	1.139	1.034	52.00	0.6039	NS
*LMO4*	0.9642	1.191	60.00	0.9044	NS
*ROCK1*	1.387	1.170	44.00	0.3561	NS
*VEGFA*	1.572	1.274	51.00	0.5692	NS

NS, non-significant; S, significant.

### Immuno-histochemistry showed different distribution patterns for CEBPB and FGFR2 in bile duct cells in CC and HB livers

**Fig 3:**
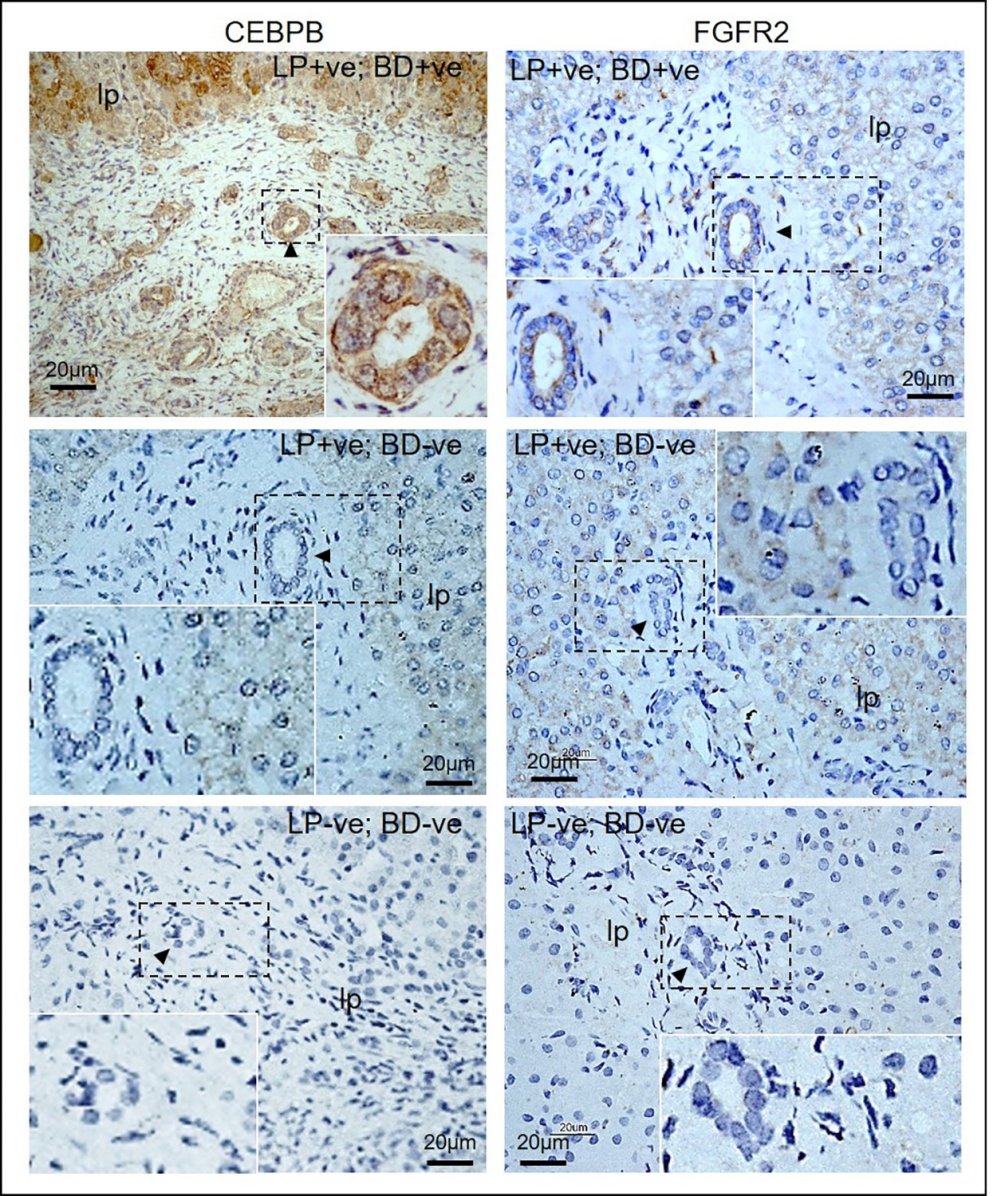
Immuno-histochemistry for FGFR2 and CEBPB in CC livers. CEBPB and FGFR2 immuno-reactivity (brown) was localized to the bile duct (arrowhead) or liver parenchyma (lp). Regions highlighted were enlarged and shown as insets.

**Fig 4:**
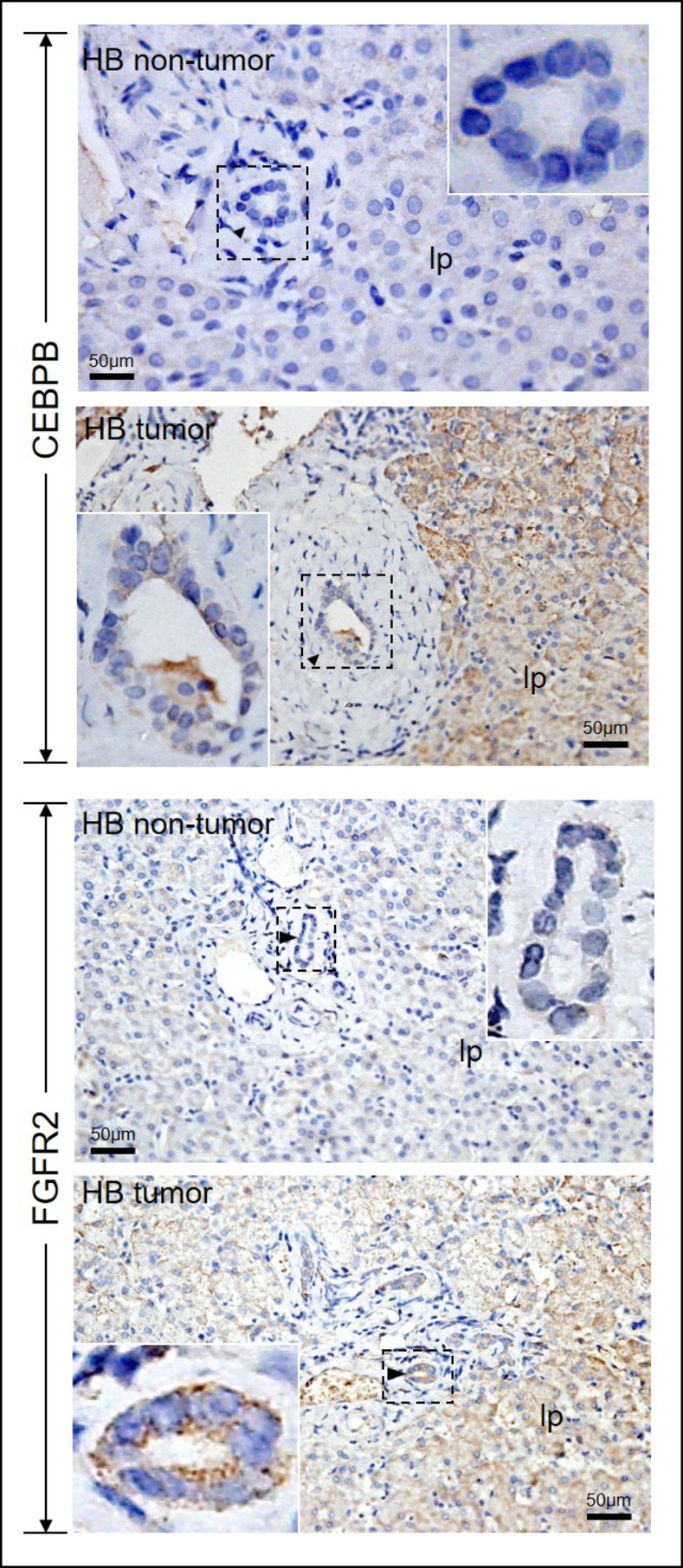
Immuno-histochemistry for FGFR2 and CEBPB in HB livers. CEBPB and FGFR2 immuno-reactivity (brown) was localized to the bile duct (arrowhead) or liver parenchyma (lp). Regions highlighted were enlarged and shown as insets.

Immuo-histochemistry revealed different patterns of CEBPB and FGFR2 expression in CC livers. CEPBB and FGFR2 expression were detected at the liver parenchyma (LP) and bile duct (BD) (LP+ve; BD+ve), or only at the liver parenchyma (LP+ve; BD-ve), or undetected (LP-ve; BD-ve) in CC livers (Fig 3). In HB non-tumor and tumor regions, similar level of expression of CEBPB and FGFR2 were localized at the liver parenchyma. In contrast, bile duct expression of CEBPB and FGFR2 were elevated in HB tumor regions as compared to the non-tumor regions (Fig 4).

To quantitate the percentages of bile duct cells that were immuno-positive for CEBPB or FGFR2 in CC and HB livers, we counted all the bile duct cells and the CEBPB immuno-positive (CEBPB+ve) or FGFR2 immuno-positive (FGFR2+ve) bile duct cells on CC (n = 35) and HB (n = 10) liver sections (tumor and non-tumor). We performed statistical analysis on the percentages of CEBPB+ve / FGFR2+ve bile duct cells / parenchymal cells between two groups, either CC vs. HB tumor group; or CC vs. HB non-tumor group. Percentages of bile duct cells that were CEBPB+ve were comparable between CC and HB tumor region, but were significantly higher than that of HB non-tumor livers (Table 2). Percentages of bile duct cells that were FGFR2+ve were significantly higher in HB tumor region than in CC and HB non-tumor livers. The percentage of bile duct cells that were FGFR2+ve in CC liver was higher than that of HB non-tumor, although it did not reach a statistical significance level. We also quantitated the percentages of parenchymal cells that were CEBPB+ve or FGFR2+ve in CC and HB tumor and non-tumor livers, and found that the percentages of CEBPB+ve and FGRF2+ve parenchymal cells were not significantly different between the three groups.

**Table 2:** Comparison of % CEBPB and FRGR2 immuno-positive bile duct cells between CC, HB tumor and non-tumor livers.

	CC	HB tumor	HB non-tumor
% CEBPB immuno-positive BD cells (Mean±S.E.M.) (No. of BD examined; No. of BD cells examined)	49.6 ± 7.0 (335; 3390); *p* = 0.3086^a^; *p* = 0.0077^b^	26.7± 4.2 (41; 584); *p* = 0.002^b^	5.2 ± 2.4 (56; 555)
% FGFR2 immuno-positive BD cells (Mean±S.E.M.) (No. of BD examined; No. of BD cells examined)	18.5 ± 4.3 (300; 3721); *p* = 0.0105^a^; *p* = 0.1522^b^	63.6 ± 0.8 (25; 242); *p* = 0.0001^b^	6.2 ± 3.6 (33; 380)

a, Comparison with HB tumor; b, Comparison with HB non-tumor.

### No significant correlations between FGFR2 and CEBPB bile duct cell immuno-reactivity and clinical parameters of CC patients

CC patients could exhibit abnormal liver function tests before surgery, like elevated level of bilirubin or liver enzymes in serum (S3 Table). Pearson correlation analysis revealed no strong correlation between FGFR2 and CEBPB bile duct cell immuno-reactivity and the age or liver function parameters (TBil, DBil, AST, ALT, γ-GT, Table 3; S4 Fig) at the time of operation.

**Table 3:** Correlation analysis of FGFR2 and CEBPB immuno-reactivity and clinical parameters.

		% immuno-positive stained bile duct cells	
		CEBPB	FGFR2
Pearson correlation	Age	-0.001883	0.03523
	TBil	0.1343	-0.1462
	DBil	0.09617	-0.1688
	ALT	0.1114	-0.1514
	AST	0.1451	-0.1827
	γ-GT	0.1930	-0.2722
Sig. (2-tailed)	Age	0.9927	0.8644
	TBil	0.5130	0.4759
	DBil	0.6630	0.4199
	ALT	0.5881	0.4605
	AST	0.4795	0.3718
	γ-GT	0.3554	0.1880

TBil: Total Bilirubin; DBil: Direct Bilirubin; ALT: Alanine Transaminase; AST: Aspartate Aminotransferase; γ-GT: Gamma-glutamyl Transpeptidase.

## Discussion

Choledochal cysts (CCs) are prone to cancer development, especially into cholangiocarcinoma (70.4%), pancreatic and gallbladder cancers (23.5%) [4–7], and the risk of cancer persists despite complete cyst removal [8]. It was found that 1/48 CC patients who underwent surgical resection developed cholangiocarcinoma [35], though it was much lower than an average malignancy transformation rate of 7.5% among those with CC [8, 36]. However, the etio-pathogenesis and molecular pathway underlying the malignant transformation of CC are not available. Genetic studies on this transformation from CC showed that *TP53*, *RBM10*, *KRAS* mutations and *FGFR2* rearrangements might trigger cancer development [37–39]. To address if CC patients’ cholangiocytes display cancer-related transcriptome signature, which may contribute to malignant transformation of CC, we grew liver organoids from CC, hepatoblastoma and (HB) (tumor and adjacent non-tumor parts), followed by RNA sequencing.

HB is an embryonal neoplasm arises from hepatic stem cells (HSC), which has a bipotential differentiation potential and give rise to both hepatic or biliary cell lineages [21]. The liver tissue-derived organoids are derived from HSC (hepatoblasts) and they are consisted of HSC, HSC-derived hepatocytes, and HSC-derived cholangiocytes as a major cell type [22–24]. 10X Genomics single-cell sequencing of our liver tissue-derived organoids indicated that common cholangiocyte markers epithelial cell adhesion molecule (EPCAM) and cytokeratin 19 (KRT19) were uniformly highly expressed in all the clusters, indicating the heterogeneity of cholangiocytes in these organoids was relatively low (S3 Fig). Transcriptome analysis revealed that HB non-tumor liver organoid clustered in a unique group separating from the tumor part. Cancer-related gene expression signature of HB tumor organoids indicates that HB tumor region HSC-derived cholangiocytes exhibits cancer features, which is in line with the reports that the differentiation of neoplastic HSC into cholangiocyte-like cells in HB [40, 41]. Intriguingly, we identified that organoids from 20–40% of CC patients displayed cancer-signature and clustered closely to HB-tumor. Hence, clustering of CC patient organoids with HB-tumor derived organoids may indicate the presence of neoplastic HSC in the livers of a subgroup of CC patients. Future single cell RNA sequencing analysis of CC and HBT livers is needed to confirm the presence of these neoplastic HSC in CC and HBT livers.

Transcriptome analysis of CC, HB tumor and HB non-tumor organoids also identified some differentially expressed (DE) genes shared a common elevated expression level in both HBT organoids and CC organoids that were closely clustered to HBT compared to HB non-tumor organoids, indicating that cancer-signature existed in some CC patients. A subsequent validation in a separate cohort of a larger series of CC and HB non-tumor livers revealed elevated expressions of *FGFR2*, *CEBPB* in CC patients, though other DE genes didn’t show differential expressions. The elevated expression of *FGFR2*, *CEBPB*, but not the other DG genes in CC livers in the subsequent validation could be attributed to the complex genetic etiologies for the cancer prone in CC, in that some DE genes (such as *FGFR2* and *CEBPB*) may have a bigger effect size on most CC patients whereas the other DE genes have effect only in a small group of CC patients.

*FGFR2* gene encodes a cell surface receptor namely fibroblast growth factor receptor 2, and it is a receptor for fibroblast growth factor. Besides playing an important role in central nervous system embryonal development, FGFR signaling influences angiogenesis and tumor cell migration, differentiation, proliferation, and survival (For a review, please see [42]). FGFR2 fusions, seen in around 13–14% of patients in intracellular cholangiocarcinoma (iCCA), have been identified as an oncogenic events driven in iCCA [43–46]. *FGFR2* was commonly elevated in bile duct cancer, especially the intrahepatic cholangiocarcinoma, and thus it is used as a marker and therapeutic target for it [37, 43, 47, 48]. *CEBPB* gene encodes a transcription factor CCAAT/enhancer-binding protein beta, that can bind as a homodimer to certain DNA regulatory regions, playing important functions in the regulation of genes involved in immune and inflammatory responses (For a review, please see [49]). Furthermore, *CEBPB* was associated with various neoplasms, including leukemia, esophageal squamous cell carcinoma, malignant breast cancer, and lung cancer [50–54] under various patho-mechanisms, such as prevention of cell death and enhancement of self-renewal potential, or as prerequisites for formation of the NRF2-dependent enhancers to enhance tumor-initiating activity [55] and thus drives malignancy in lungs [51]. In line with the presence of cholangiocyte-like cells in HB [40, 41], and elevated expression of *FGFR2* in bile duct cancer [37, 43, 47, 48], percentages of FGFR2-immuno-positive bile duct cells were the highest in HB tumor region as compared to CC and HB non-tumor livers. Notably, the percentage of FGFR2-immuno-positive bile duct cells in CC liver was also higher than that of HB non-tumor. Percentages of CEBPB-immuno-positive bile duct cells were comparable between CC and HB tumor region, but both were significantly higher than that of HB non-tumor livers. In contrast, the percentages of parenchymal cells that were immuno-positive for CEBPB or FGFR2 were not significantly different between CC and HB tumor and non-tumor livers. Our data showed that the elevation of FGFR2 and CEBPB in HB-tumor and in a subgroup of CC patients are restricted to bile duct only, and *CEBPB* and *FGFR2* was markedly over-expressed in 6.5% (2 of the 31) and 22.6% (7 of the 31) CC patients, respectively. Although, most of the bile duct cancers arising in CC locate in the gallbladder or the extrahepatic bile ducts, and intrahepatic cholangiocarcinoma is relatively rare. Genomic characterization and tissue microarray studies of bile duct cancers have revealed common genomic aberrations and dysregulated expression profiles in intra- and extrahepatic cholangiocarcinoma, which suggests that intra- and extrahepatic bile duct cancers share some common pathobiological pathways [56, 57]. Cancer therapies targeting some of these common pathways are effective for both intra- and extrahepatic bile duct cancers (For a review, see [58]). Indeed, high level expressions of CEBPB are also detected in the liver parenchyma and intrahepatic bile ducts of patients with bile duct cancer (The Human Protein Atlas; https://www.proteinatlas.org/ENSG00000172216-CEBPB/pathology/liver+cancer#ihc). However, the role of CEBPB in cancers is controversial and CEBPB can display both pro- and anti-tumorigenic activities, depending on different cell/tissue context; different causes of the cancers, such as homo- or—heterodimerization, presence of inhibitors or presence of different isoforms of CEBPB (For a review, please see [59]). Further study is needed to investigate the expression profile of different CEBPB isoforms, post-translation activation of CEBPB by phosphorylation, expression of CEBPB activity activators and inhibitors in neoplastic and non-neoplastic hepatoblastoma tissues and CC tissues will clarify the contribution of CEBPB in hepatoblastoma and cancer development in CC. Furthermore, it is needed to address if elevation of FGFR2 and CEBPB are also similarly found in the gallbladder and extrahepatic bile duct cancers in CC patients.

Taken all the above indicated that CEBPB and FGFR2 were differentially elevated in the bile duct cells of a sub-group of CC patients, and that elevated expression of FGFR2 and CEBPB may contribute to cancer development in CC. A long-term follow up of this subgroup of CC patients is needed to confirm if elevated expression of CEBPB and FGFR2 is associated with the higher incidence of bile duct cancer in these patients.

## Limitations

We acknowledge that there are several limitations in our study. First, we do not have a long follow-up of these enrolled CC patients to see whether patients with high level of *FGFR2* and *CEBPB* were more prone to develop into tumor. Second, our study does not include the CC with dysplasia and carcinoma group, and also the mechanisms such as anomalous pancreaticobiliary junction and pancreatobiliary reflux are not taken into consideration on the effect on malignancy development, which has affected the analysis of the true clinical implications.

## Conclusions

CC has a potential to develop into bile duct cancer, without biomarker and due to the asymptomatic characteristics of the cancer, the cancer is diagnosed at the advanced stage of malignancy with very poor prognosis. Future study to address the efficacy of *FGFR2* and *CEBPB* as neo-biomarkers for cancer risk stratification of CC patients and drug targets for bile duct cancer treatment in a larger patient cohort with long-term follow up is warranted.

## Supporting information

S1 Fig Human liver organoid cells expressed markers of cholangiocyte and hepatoblast. (A) UMAP showing the clusters in 10X Genomics single-cell RNA sequencing analysis of human liver organoids showing the assigned identity for each cluster (3 clusters) (B) The total number of cells per cluster in bar plot in human liver organoids. (C) Violin plot showing expression of canonical hepatobiliary markers used for identification of clusters including cytokeratin-19 (KRT19) and epithelial cellular adhesion molecule (EPCAM).
(TIF)

S2 Fig Differential expression and pathway analysis. (A, B, C). Non-linear dimensional reduction (tSNE) showing clustering of patient derived organoid with sample identifiers for each comparisons. (D, E, F) Volcano plot showing dysregulated genes for each comparisons. (G) Venn diagram showing comparison of transcriptome analysis based on conditions. (H, I, J) Pathway analysis for up-regulated and down-regulated genes in comparisons.
(TIF)

S3 Fig Clustering and pathway analysis from patient derived organoids. (A) Principal Component Analysis (PCA) analysis showing first two PCs capturing highest variance, 69% (dimension 1: 60% + dimension 2: 9%) in expression data with sample identifiers; each dot represents an organoid. (B) Non-linear dimensional reduction (tSNE) showing clustering of patient derived organoid with sample identifiers. (C) Pathway analysis for up-regulated and down-regulated genes in CC when compared to HB and HB-Tumor. (D) Significantly enriched pathways for DE genes using KEGG pathway analysis.
(TIF)

S4 Fig No strong correlation between FGFR2 and CEBPB bile duct cell immuno-reactivity with clinical parameters of CC patients. Pearson correlation analysis was performed for FGFR2 and CEBPB bile duct cell immuno-reactivity with the age or liver function parameters at the time of operation.
(TIF)

S1 Table Primers for RT-qPCR expression validation of genes of human liver tissues. (DOCX)

S2 Table Primary and secondary antibodies used in the IHC experiment. (DOCX)

S3 Table Clinical information of CC patients with significantly elevated FGFR2 & CEBPB. (DOCX)

S4 Table Clinical information of CC patients included in the study. (DOCX)

S5 Table Clinicopathological features of HB samples used in the study. (DOCX)

## Abbreviations

CC: Choledochal cyst
HB: hepatoblastoma
FGFR2: fibroblast growth factor receptor 2
CEBPB: CCAAT/enhancer-binding protein beta
RNAseq: RNA sequencing
