# Supplementary figures and images for: Identification of cancer-related genes FGFR2 and CEBPB in choledochal cyst via RNA sequencing of patient-derived liver organoids
Source: PLoS One. 2023 Mar 30;18(3):e0283737. doi: 10.1371/journal.pone.0283737 (PMC10062558; doi:10.1371/journal.pone.0283737)

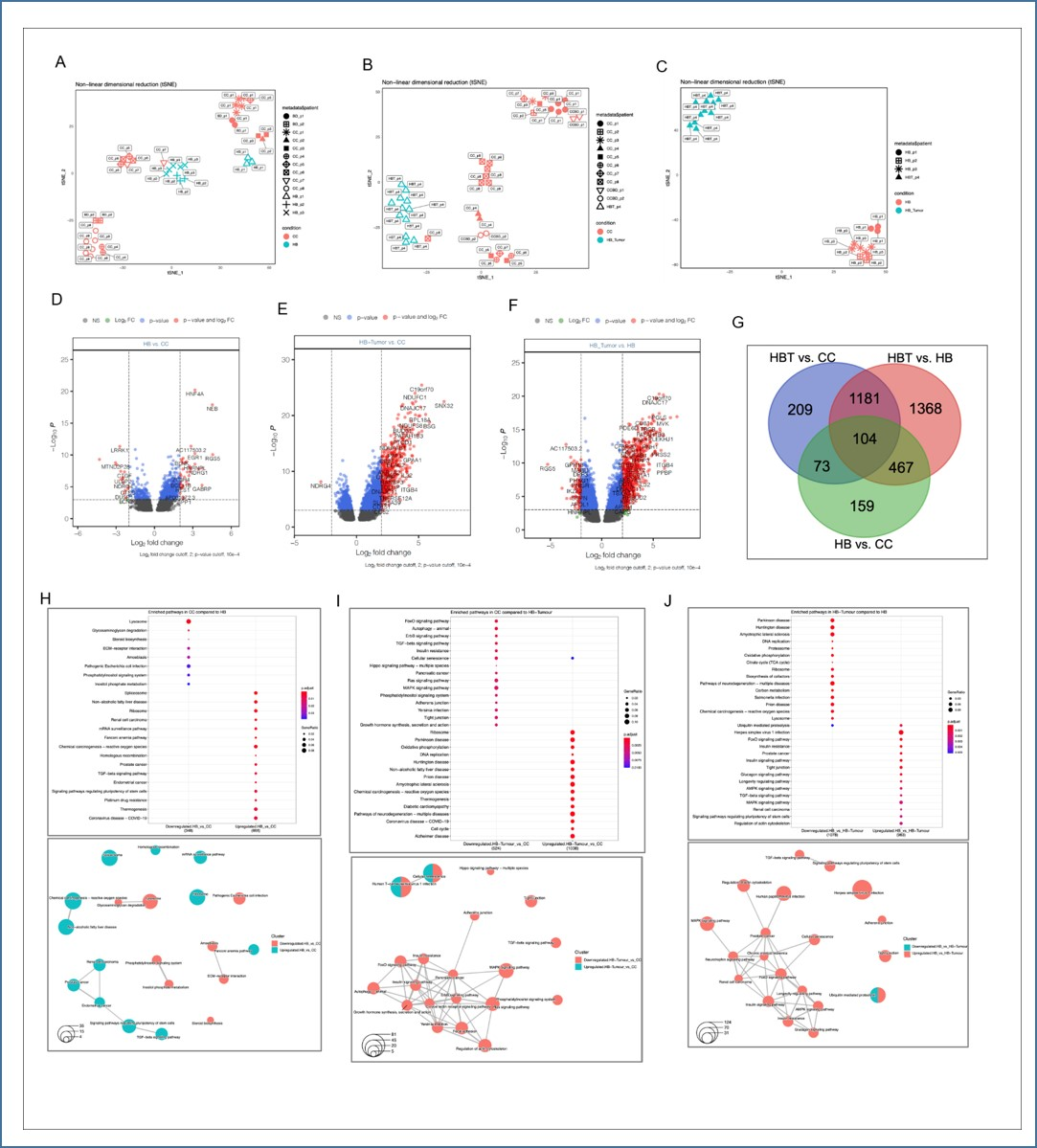

Supplement: S1 Fig — (A) UMAP showing the clusters in 10X Genomics single-cell RNA sequencing analysis of human liver organoids showing the assigned identity for each cluster (3 clusters) (B) The total number of cells per cluster in bar plot in human liver organoids. (C) Violin plot showing expression of canonical hepatobiliary markers used for identification of clusters including cytokeratin-19 (KRT19) and epithelial cellular adhesion molecule (EPCAM). (TIF) [file pone.0283737.s001.tif]

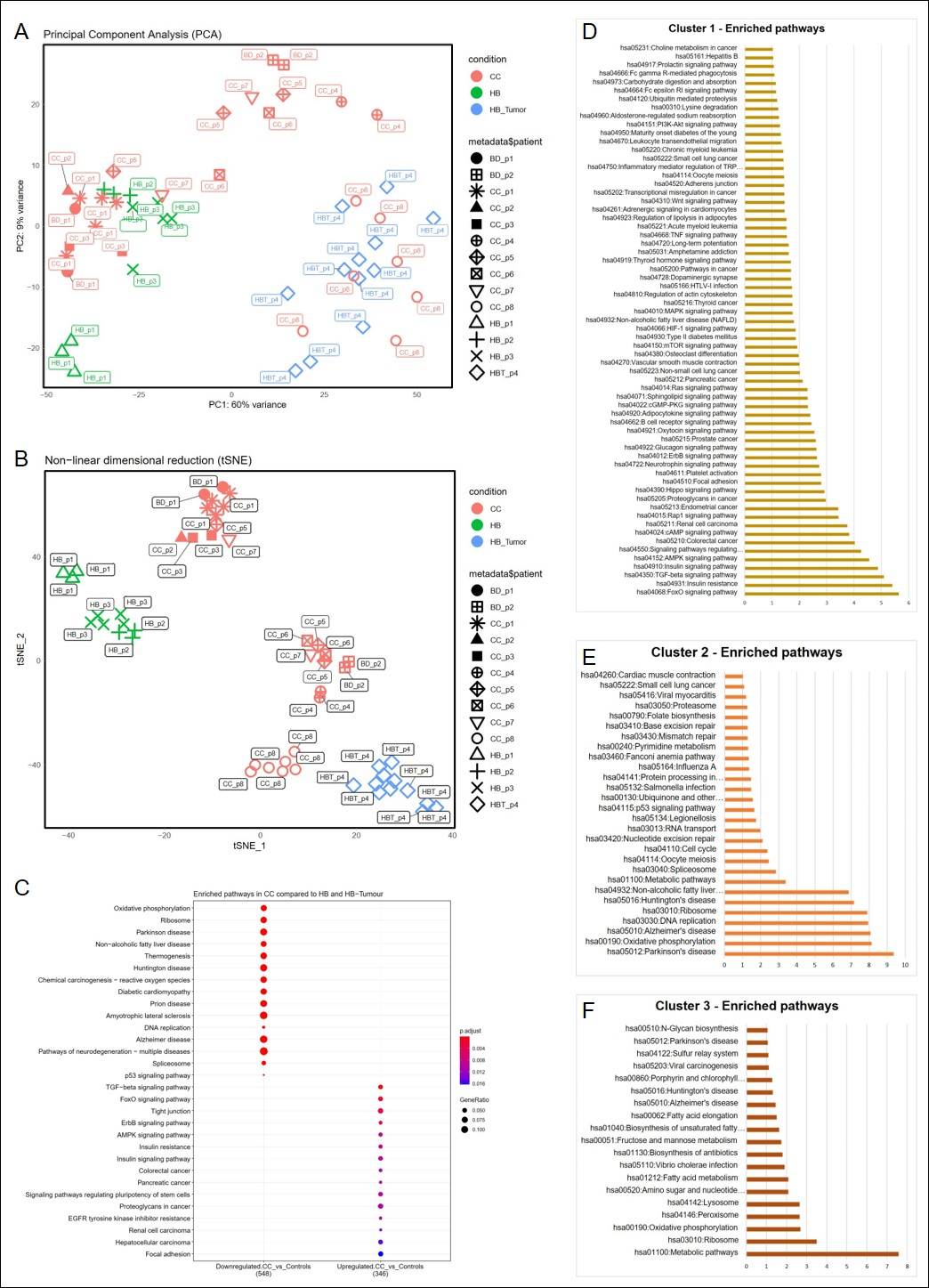

Supplement: S2 Fig — (A, B, C). Non-linear dimensional reduction (tSNE) showing clustering of patient derived organoid with sample identifiers for each comparisons. (D, E, F) Volcano plot showing dysregulated genes for each comparisons. (G) Venn diagram showing comparison of transcriptome analysis based on conditions. (H, I, J) Pathway analysis for up-regulated and down-regulated genes in comparisons. (TIF) [file pone.0283737.s002.tif]

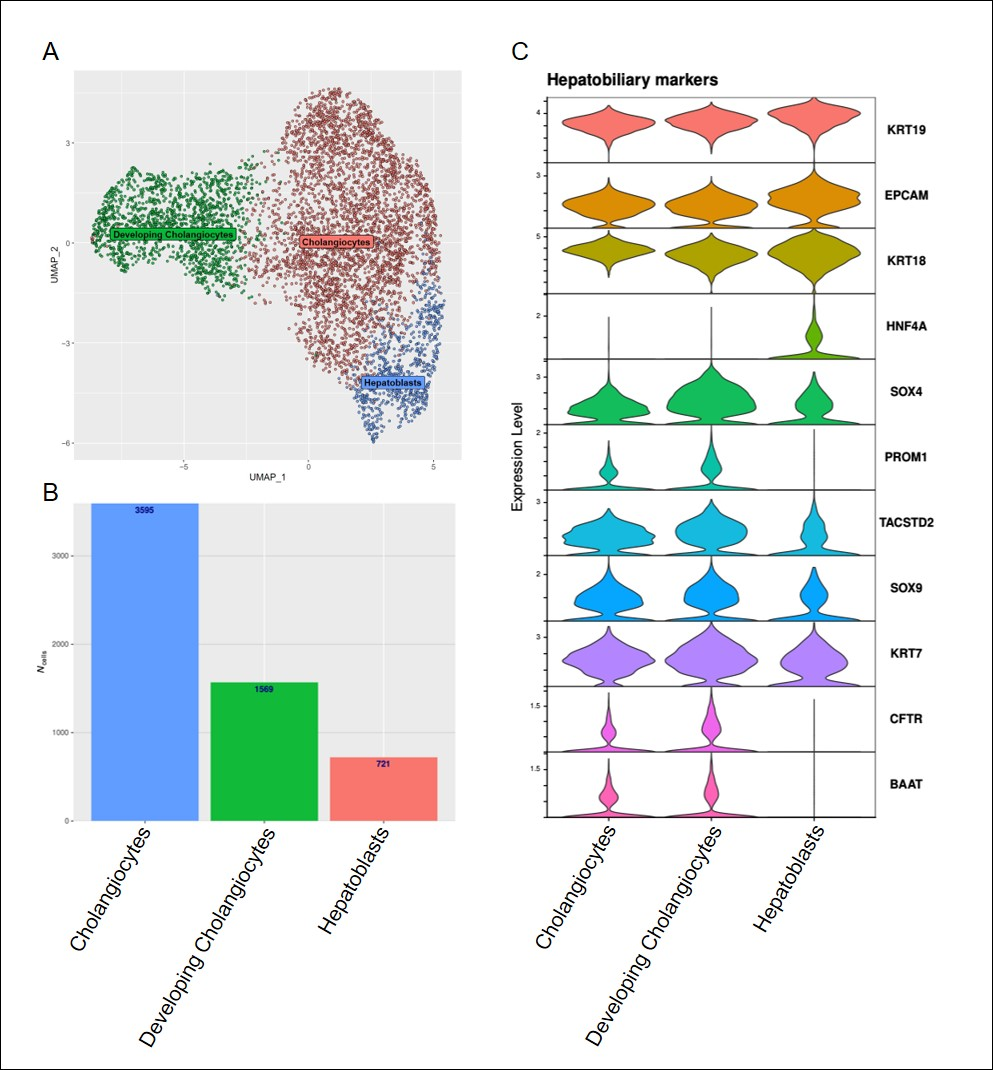

Supplement: S3 Fig — (A) Principal Component Analysis (PCA) analysis showing first two PCs capturing highest variance, 69% (dimension 1: 60% + dimension 2: 9%) in expression data with sample identifiers; each dot represents an organoid. (B) Non-linear dimensional reduction (tSNE) showing clustering of patient derived organoid with sample identifiers. (C) Pathway analysis for up-regulated and down-regulated genes in CC when compared to HB and HB-Tumor. (D) Significantly enriched pathways for DE genes using KEGG pathway analysis. (TIF) [file pone.0283737.s003.tif]

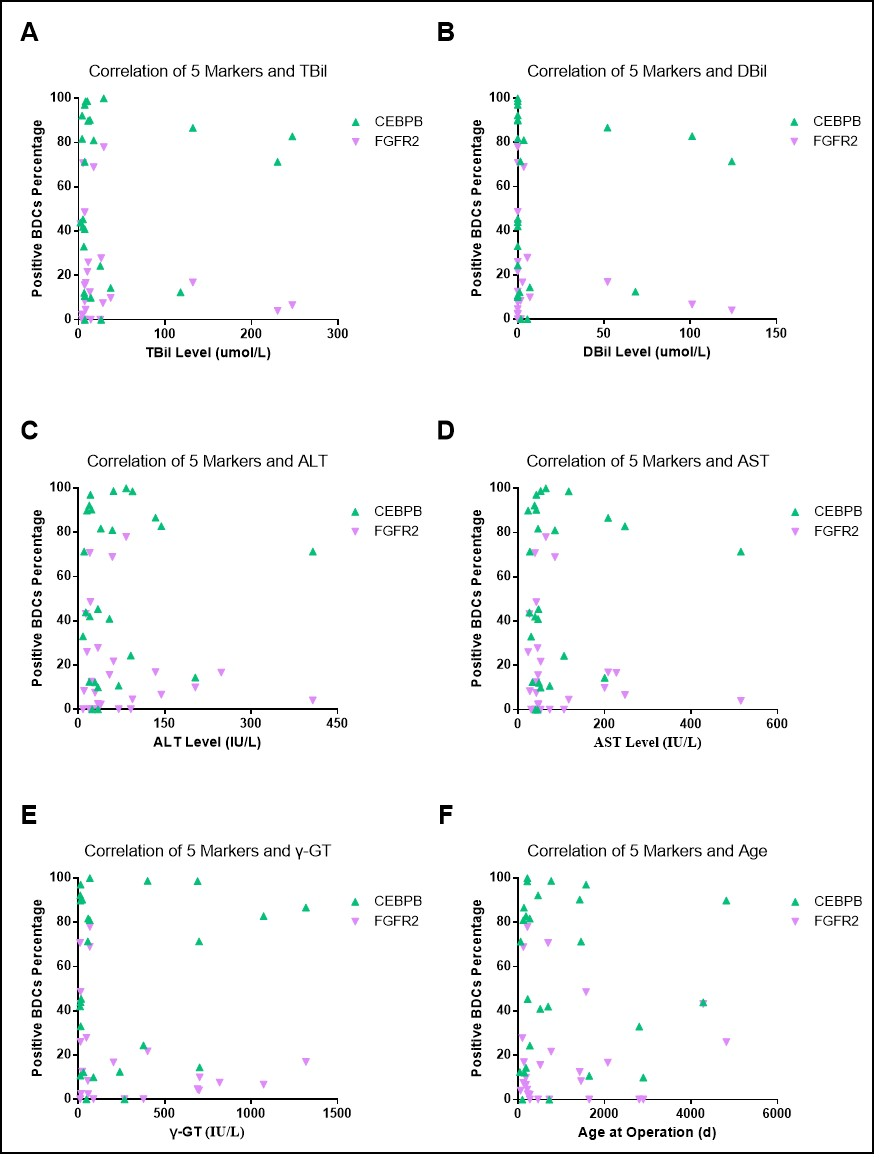

Supplement: S4 Fig — Pearson correlation analysis was performed for FGFR2 and CEBPB bile duct cell immuno-reactivity with the age or liver function parameters at the time of operation. (TIF) [file pone.0283737.s004.tif]
