# Supplementary material for: Identification of cancer-related genes FGFR2 and CEBPB in choledochal cyst via RNA sequencing of patient-derived liver organoids
Source: PLoS One. 2023 Mar 30;18(3):e0283737. doi: 10.1371/journal.pone.0283737 (PMC10062558; doi:10.1371/journal.pone.0283737)
Supplement: S1 Table — (DOCX) [file pone.0283737.s005.docx]

**S1 Table. Primers for RT-qPCR expression validation of genes of human liver tissues**

| Gene | Primers |
| --- | --- |
| *ABCA5* | Forward: GCTTGCCTTTCAAGGACATGGAC  Reward: CCAAAGTCGTCATGGAAACACCA |
| *CDKN1B* | Forward: ATAAGGAAGCGACCTGCAACCG  Reward: TTCTTGGGCGTCTGCTCCACAG |
| *CEBPB* | Forward: AGAAGACCGTGGACAAGCACAG  Reward: CTCCAGGACCTTGTGCTGCGT |
| *FGFR2* | Forward: GTGCCGAATGAAGAACACGACC  Reward: GGCGTGTTGTTATCCTCACCAG |
| *FLCN* | Forward: GATTGAAGCGGCTCTGACCAAC  Reward: TCGACTGTCCACCTTGGTGAAC |
| *FZD1* | Forward: GCTTTGTGTCGCTCTTCCGCAT  Reward: TACAGCACGCTGAAGACGCCAA |
| *LPAR6* | Forward: GCTCAGTAGTGGCAGCAGTAAG  Reward: GTCACTTCTCCTGACAGACCAG |
| *LMO4* | Forward: CAGTCGATTCCTGCGAGTGAAC  Reward: CTGCCATTGATGTAGTGAAACCG |
| *ROCK1* | Forward: GAAACAGTGTTCCATGCTAGACG  Reward: GCCGCTTATTTGATTCCTGCTCC |
| *VEGFA* | Forward: TTGCCTTGCTGCTCTACCTCCA  Reward: GATGGCAGTAGCTGCGCTGATA |
| *GAPDH* | Forward: GTCTCCTCTGACTTCAACAGCG  Reward: ACCACCCTGTTGCTGTAGCCAA |
