# Supplementary material for: Identification of cancer-related genes FGFR2 and CEBPB in choledochal cyst via RNA sequencing of patient-derived liver organoids
Source: PLoS One. 2023 Mar 30;18(3):e0283737. doi: 10.1371/journal.pone.0283737 (PMC10062558; doi:10.1371/journal.pone.0283737)
Supplement: S2 Table — (DOCX) [file pone.0283737.s006.docx]

**S2 Table. Primary and secondary antibodies used in the IHC experiment**

| Antigen | Primary antibody | Secondary Antibody | Antigen retrieval buffer |
| --- | --- | --- | --- |
| ABCA5 | Anti-ABCA5 antibody (abcam; ab99953); Rabbit polyclonal, (1:8000) | EnVision+ System- HrP Labelled Polymer Anti-Rabbit (Dako; 11199901) | 10mM sodium citrate buffer (pH 6.0) |
| CEBPB | Anti- CEBP Beta antibody (abcam; ab264305); Rabbit polyclonal, (1:10000) |  |  |
| FGFR2 | Anti-Fibroblast Growth Factor Receptor-2 (Sigma-Aldrich; F0300); Rabbit polyclonal, (1:300) |  |  |
| LMO4 | Anti-LMO4 antibody (abcam; ab251966); Rabbit polyclonal, (1:50) |  |  |
| LPAR6  (P2RY5) | Anti- P2RY5 antibody (abcam; ab272600); Rabbit polyclonal, (1:30) |  |  |
