# Supplementary material for: Identification of cancer-related genes FGFR2 and CEBPB in choledochal cyst via RNA sequencing of patient-derived liver organoids
Source: PLoS One. 2023 Mar 30;18(3):e0283737. doi: 10.1371/journal.pone.0283737 (PMC10062558; doi:10.1371/journal.pone.0283737)
Supplement: S3 Table — (DOCX) [file pone.0283737.s007.docx]

**S3 Table. Clinical information of CC patients with significantly elevated *FGFR2* & *CEBPB***

| Patient | LFTs | | | | | Fold change vs. HB (*CEBPB*) | Fold change vs. HB (*FGFR2*) |
| --- | --- | --- | --- | --- | --- | --- | --- |
|  | TBil | DBil | ALT | AST | γ-GT |  |  |
| 1 | 29.4 | 10.9 | 29 | 44 | 59 | 1.4054 | 8.1275 |
| 2 | 50.2 | 13.6 | 115 | 110 | 332 | 5.8690 | 5.2315 |
| 3 | 5.8 | 1.0 | 43 | 55 | 34 | 3.5526 | 7.8796 |
| 4 | 28.9 | 11.9 | 178 | 92 | 503 | 13.2868 | 8.3683 |
| 5 | 14.9 | 2.0 | 59 | 73 | 184 | 21.0538 | 6.8562 |
| 6 | 10.0 | 2.0 | 23 | 38 | 36 | 3.6142 | 5.1214 |
| 7 | 7.5 | 1.8 | 36 | 54 | 455 | 2.8230 | 11.5428 |

TBil: Total Bilirubin; DBil: Direct Bilirubin; ALT: Alanine Transaminase; AST: Aspartate Aminotransferase; γ-GT: Gamma-glutamyl Transpeptidase.
