# Supplementary material for: Identification of cancer-related genes FGFR2 and CEBPB in choledochal cyst via RNA sequencing of patient-derived liver organoids
Source: PLoS One. 2023 Mar 30;18(3):e0283737. doi: 10.1371/journal.pone.0283737 (PMC10062558; doi:10.1371/journal.pone.0283737)
Supplement: S4 Table — (DOCX) [file pone.0283737.s008.docx]

**S4 Table. Clinical information of CC patients included in the study**

| **Patient no.** | **Gender** | **Abdominal pain due to biliary pancreatitis (YES or NO, if yes, how many times it occurred before surgery)** | **Jaundice (YES or NO)** | **TBIL** | **DBIL** | **IBIL** | **ALT** | **AST** | **γ-GT** | **ALP** | **Type** |
| --- | --- | --- | --- | --- | --- | --- | --- | --- | --- | --- | --- |
| 19-32266-B | F | NO | YES | 247 | 101 | 36 | 144 | 247 | 1072 | 798 | 1 |
| 19-27962-B | F | NO | NO | 4 | 0 | 2 | 19 | 38 | 11 | 288 | 1 |
| 19-15858-A | F | NO | NO | 4 | 0 | 3 | 39 | 46 | 57 | 148 | 1 |
| 18-9324 | F | NO | YES | 10 | 0 | 1 | 61 | 52 | 401 | 551 | 1 |
| 18-3157-A | F | YES | NO | 13 | 0 | 6 | 23 | 42 | 23 | 214 | 1 |
| 18-28493 | F | YES | NO | 7 | 0 | 2 | 21 | 42 | 14 | 225 | 1 |
| 17-25670-A | F | YES | NO | 11 | 0 | 5 | 15 | 23 | 13 | 125 | 1 |
| 17-11449-C | M | YES | NO | 2 | 0 | 2 | 13 | 26 | 13 | 163 | 4 |
| 17-10348 | F | NO | YES | 5 | 0 | 3 | 20 | 39 | 10 | 286 | 1 |
| 16-29707 | F | NO | NO | 5 | 0 | 0 | 34 | 47 | 16 | 181 | 1 |
| 16-19918-A | M | NO | NO | 6 | 0 | 2 | 8 | 30 | 14 | 222 | 1 |
| 15-24221 | F | NO | YES | 132 | 52 | 24 | 134 | 208 | 1317 | 386 | 1 |
| 13-34049 | F | NO | NO | 8 | 0 | 4 | 94 | 117 | 690 | 218 | 1 |
| 13-10663 | F | NO | YES | 29 | 0 | 11 | 83 | 64 | 67 | 965 | 1 |
| 12-7881 | F | NO | NO | 10 | 0 | 5 | 85 | 84 | 202 | 384 | 1 |
| 12-34266-B | F | NO | NO | 230 | 124 | 40 | 407 | 515 | 699 | 335 | 1 |
| 12-31874-B | F | NO | NO | 37 | 7 | 4 | 203 | 200 | 702 | 330 | 1 |
| 12-25148-D | M | YES, 1 | YES | 7 | 0 | 0 | 70 | 73 | 12 | 154 | 1 |
| 11-4867 | F | NO | YES | 28 | 0 | 8 | 29 | 42 | 818 | 835 | 1 |
| 11-29203-C | F | NO | NO | 3 | 0 | 3 | 18 | 39 | 17 | 220 | 1 |
| 10-19352-B | F | YES, 1 | NO | 24 | 0 | 8 | 50 | 39 | 82 | 158 | 1 |
| 08-8601 | M | YES, 1 | YES | 14 | 0 | 10 | 34 | 52 | 88 | 248 | NA |
| 07-16791 | F | NO | NO | 25 | 0 | 9 | 91 | 106 | 377 | 1206 | 4 |
| 04-24182-B | F | YES, 1 | NO | 7 | NA | NA | 54 | 46 | NA | 273 | 4 |
| 387875 | M | NO | YES | 117.9 | 68.2 | 49.7 | 19 | 33 | 240 | 569 | 1 |
| 403584 | M | NO | NO | 26.1 | 5.6 | 20.5 | 34 | 45 | 47 | 240 | 1 |
| 353892 | F | NO | NO | 17.4 | 3.5 | 13.9 | 59 | 85 | 66 | 253 | 1 |
| 387707 | F | NO | NO | 6.5 | 1.1 | 5.4 | 29 | 48 | 30 | 249 | 1 |
| 392968 | M | YES | NO | 8.2 | 2.8 | 5.4 | 248 | 227 | 204 | 385 | 4 |
| 393363 | F | YES | NO | 7.2 | 1.9 | 5.3 | 24 | 40 | 268 | 319 | 1 |
| 403850 | F | YES | NO | 7 | 1.6 | 5.4 | 10 | 27 | 55 | 253 | 1 |
| 390253 | M | NO | NO | 87.3 | 45 | 42.3 | 44 | 75 | 103 | 437 | 1 |

TBil: Total Bilirubin; DBil: Direct Bilirubin; IBil: Indirect Bilirubin; ALT: Alanine Transaminase; AST: Aspartate Aminotransferase; γ-GT: Gamma-glutamyl Transpeptidase; ALP: ; Alkaline Phosphatase.
