# Supplementary material for: Identification of cancer-related genes FGFR2 and CEBPB in choledochal cyst via RNA sequencing of patient-derived liver organoids
Source: PLoS One. 2023 Mar 30;18(3):e0283737. doi: 10.1371/journal.pone.0283737 (PMC10062558; doi:10.1371/journal.pone.0283737)
Supplement: S5 Table — (DOCX) [file pone.0283737.s009.docx]

**Table S5 Clinicopathological features of HB samples used in the study**

| **Sample** | **Subtype** | **Age**  **(month)** | **Sex** | **Tumour size** | **Preoperative chemotherapy** | **Resection margin state** | **Clinical course** |
| --- | --- | --- | --- | --- | --- | --- | --- |
| 1 | Fetal | 16 | F | 3.9x3.1x2cm | Yes | Clear (1mm) | No recurrence |
| 2 | Mixed epithelial and mesenchymal type | 24 | M | 4cm | Yes | Clear (1mm) | No recurrence |
| 3 | Fetal epithelial differentiation | 38 | F | 6.5X1.5X6.5cm | Yes | Clear (5mm) | No recurrence |
| 4 | Embryonal | 48 | F | 6cm | Yes | Not applicable | No recurrence |
| 5 | Embryonal with a minor component of fetal type | 62 | F | Tumour 1: 1-1.4cm; Tumour 2: 1.5-2cm | Yes | Tumour 1: not involved; Tumour 2: involved | Poor, relapse and die for terminal malignancy |
| 6 | Epithelial type | 55 | F | 8x6x10cm | Yes | Clear (2mm) | No recurrence |
| 7 | Epithelial type with a fetal pattern | 71 | M | 6cm | Yes | Clear (1mm) | No recurrence |
| 8 | No mention | 32 | F | Multifocal: 1.3cm, 2.5cm, 1.6cm, 0.2cm | Yes | Not applicable | No recurrence |
| 9 | Pure fetal epithelial | 84 | F | 9.2x7.5x7.5cm | Yes | Clear (1.5mm) | Poor, die for terminal malignancy |
| 10 | Embryonal | 57 | F | Not available | Yes | Not involved | No recurrence |
| 11 | Pure epithelial, mixed fetal and embryonal | 43 | M | 9.4x9.7x10cm | No | Not involved | Poor |
| 12 | Pure epithelial, mixed fetal and embryonal | 27 | M | 10cm | No | Not involved | Poor |
| 13 | Fetal and embryonal | 48 | M | Not available | Yes | Clear (1mm) | No recurrence |
| 14 | Fetal | 24 | F | 4cm | Yes | Clear (1mm) | No recurrence |
| 15 | Mixed epithelial and embryonal | 33 | M | 4cm | Yes | Clear (3mm) | No recurrence |
